# Co-Delivery of Curcumin and Bioperine via PLGA Nanoparticles to Prevent Atherosclerotic Foam Cell Formation

**DOI:** 10.3390/pharmaceutics13091420

**Published:** 2021-09-08

**Authors:** Sindhu C. Pillai, Ankita Borah, Minh Nguyen Tuyet Le, Hiroaki Kawano, Kouichi Hasegawa, D. Sakthi Kumar

**Affiliations:** 1 Bio-Nano Electronics Research Centre, Graduate School of Interdisciplinary Science, Toyo University, 2100, Kujirai, Saitama, Kawagoe 350-8585, Japan; sindhu.09@gmail.com (S.C.P.); ankitaborah24@gmail.com (A.B.); 2 Institute for Integrated Cell-Material Sciences (iCeMS), Kyoto University, Kyoto 606-8302, Japan; le.minhnguyentuyet.8m@kyoto-u.ac.jp (M.N.T.L.); kawano.hiroaki@fbri.org (H.K.); hasegawa.kouichi.2c@kyoto-u.ac.jp (K.H.)

**Keywords:** atherosclerosis, foam cells, curcumin, bioperine, macrophages, PLGA nanoparticle

## Abstract

Cholesterol-rich arterial plaques characterize atherosclerosis, a significant cause of heart disease. Nutraceuticals have received attention over the years, demonstrating potential benefits towards treating and preventing cardiovascular diseases (CVD), including atherosclerosis. Curcumin, a potent polyphenol present in *Curcuma longa*, has shown remarkable anti-atherosclerotic activity via anti-inflammatory and anti-oxidative properties. The bioavailability and low water solubility of curcumin limit its clinical translational purposes. These issues can be circumvented effectively by nano-drug delivery systems that can target atherosclerotic plaque sites. In this work, we chose to use curcumin and a natural bioenhancer called Bioperine (derived from *Piper nigrum*) inside a polymeric nano-drug delivery system for targeting atherosclerotic plaque sites. We selected two different ratios of curcumin:Bioperine to study its comparative effect on the inhibition of oxidized low-density lipoprotein (Ox-LDL)-induced foam cell formation. Our studies demonstrated that Cur-Bio PLGA NPs (both ratios) maintained the cell viability in THP-1 monocyte-derived macrophages above 80% at all periods. The 1:0.2:10 ratio of Cur-Bio PLGA NPs at a concentration of 250 μg/mL illustrated an enhanced reduction in the relative cholesterol content in the THP-1-derived foam cells compared to the 1:1:10 ratio. Confocal microscopy analysis also revealed a reduction in macrophage-mediated foam cell formation when administered with both the ratios of Cur-Bio PLGA NPs. Relative fold change in the mRNA expression of the genes involved in the inflammatory pathways in the atherosclerotic process downregulated NF-κB, CCL2/MCP-1, CD-36, and STAT-3 activity while upregulating the SCAR-B1 expression when treated with the Cur-Bio PLGA NPs. This study thus highlights the importance of natural-based compounds towards the therapeutic intervention against atherosclerotic activity when administered as preventive medicine.

## 1. Introduction

Atherosclerosis, being an immunoinflammatory disease, is recognized as one of the most widespread causes of cardiovascular and peripheral arterial diseases that affect the mortality rate of millions worldwide [1,2]. Atherosclerosis leads to lipid retention inside the susceptible sites of the arteries, which narrows the coronary arteries, thereby mitigating the flow of oxygen-enriched blood to the vital organs. This process leads to heart attacks, strokes, and peripheral vascular disease, collectively known as cardiovascular disease (CVD) [3,4]. The endothelium is a thin layer of lining found beneath these blood vessels that acts as a smoothening agent and ensures the smooth flow of blood. Atherosclerosis begins with endothelium dysfunction, which ultimately leads to plaque formation. The interaction between the endothelial proteoglycans and apo B-containing lipoproteins initiates low-density lipoprotein (LDL) into the walls of the arteries [5]. Reactive oxygen species (ROS), lipoxygenase (Lox), myeloperoxidase (MPO), along with the inducible nitric oxide synthase (iNOS), oxidizes the intima LDL [6]. This oxidized LDL (Ox-LDL) acts as a pro-inflammatory mediator, which initiates monocyte recruitment in the blood vessel wall where the monocytes differentiate into macrophages in response to the monocyte colony-stimulating factor (M-CSF). This monocyte differentiation process upregulates the scavenger receptor A(SR-A1) and CD-36 expression that aids in recognizing and internalizing the Ox-LDL, leading to macrophage-mediated foam cell formation [7]. Experimental pieces of evidence have elucidated that the combined inhibition of SR-A1 and CD-36 blocks foam cell formation in human and murine cells [8]. Febbraio M. et al. have shown that CD-36 accounts for most of the Ox-LDL uptake by the macrophages that lead to foam cell formation [9].

During the initiation of the atheroma stage, endothelial–leukocyte adhesion molecule has emerged as a desirable candidate for the early adhesion of mononuclear leukocytes to arterial endothelium sites. Vascular cell adhesion molecule (VCAM-1) attracts macrophages towards the endothelial lesion-prone areas [10]. In addition to VCAM-1, P- and E-selectin also play a pivotal role in monocyte recruitment [11]. Mounting evidence has shown that the involvement of nuclear factor-κB (NF-κB) regulation in the activation of pro-inflammatory cytokines such as interleukin (IL)-1β, tumor-necrosis factor-α (TNF-α) and IL-6 (interleukin-6) that induce the activation of VCAM-1 [12,13]. Current treatment approaches towards atherosclerosis involve using small-molecule drugs and pro-inflammatory cytokine inhibitors that reduce LDL and cytokine levels, respectively. Amidst all such developments, cardiovascular disease remains one of the leading causes of death worldwide, and the potential of such therapies and their direct side effects remains controversial.

An adept solution identified as a complementary therapy for cardiovascular disease resorts to developing an effective strategy to reduce inflammation before fatty streak formation. Curcumin, being a natural polyphenol that belongs to curcuminoid, is derived from the rhizomes of *Curcuma longa* that exhibit various biological and pharmacological properties encompassing anti-atherosclerotic [14] and anti-oxidant [15] properties, LDL oxidation prevention, reducing blood cholesterol, and preventing myocardial infarction via diverse molecular targets [16]. One of the significant alluring aspects of curcumin is the possession of pleiotropic effects in inducing anti-inflammatory actions by downregulating NF-κB and STAT-3 pathways [17]. The downregulation of NF-κB and STAT-3 leads to reduced VCAM-1 expression and inhibits pro-inflammatory cytokines IL-1β, TNF-α, IL-6, monocyte chemoattractant protein-1(MCP-1), CD-36, and matrix metalloproteinases (MMPs) [5]. Curcumin demonstrates elevation of the scavenger receptor class B type (SCAR-B1/SR-B1) gene expression and suppression of the c-Jun N-terminal kinase pathway (JNK) pathway, which assists in the removal of accumulated cholesterol from the macrophages [18]. Despite having such prodigious properties, the application of curcumin is minimal due to its hydrophobic nature, low aqueous solubility, poor absorption, and rapid metabolism in the body. Using an appropriate nano-drug delivery system is one way to solve the challenges faced in curcumin biomedical applications [19]. 

The appealing nature of nanoparticles in providing selective drug delivery to the cells and tissues while minimizing off-target effects has leverage over the conventional therapies currently in practice for vascular diseases. Curcumin delivery via such innovative nanocarriers has been widely studied against cancer therapies and neurodegenerative disorders in the past [20,21,22]. Previous studies from our group have reported the superior anti-cancer efficacy of curcumin nanoparticles against breast cancer [23] and pancreatic cancer [24]. The anti-oxidative and anti-amyloid properties of Cur-PLGA NPs were also investigated as a potential treatment strategy in Alzheimer’s disease, thus reporting the exceptional beneficial use of curcumin against neurodegenerative disorders [25]. The never-ending applications of curcumin nanoparticles have been explored minimally for vascular diseases, especially atherosclerosis. One of the current reports to come out in this direction of anti-atherosclerotic therapeutic strategy is the development of curcumin-loaded linear-dendrimer type methoxy-poly (ethylene glycol)-b-poly (e-caprolactone) copolymer nanoparticles, demonstrating the better efficacy in reducing atherosclerotic lesions and plaque-stabilizing properties compared to free curcumin [26]. 

The idea of incorporating a natural bio-enhancer alongside curcumin nano-delivery and to analyze the curcumin enhancement in its therapeutic efficacy against the foam cell formation in atherosclerosis was the motivation and consequently signified the novelty behind this current work. Natural bio-enhancers are agents that potentially increase the effectiveness and bioavailability of drugs when co-administered [27]. Bioperine/piperine, a naturally occurring alkaloid derived from *Piper nigrum* is a potent bioavailability enhancer due to its rapid absorption characteristics and the ability to inhibit the metabolization of enzymes solely responsible for biotransformation of drugs [28,29]. Owing to the hydrophobicity and low bioavailability of Bioperine, its dosage requirement in being used as a bio-enhancer restricts its clinical translation. Henceforth, this impediment’s possible solution would be to incorporate it into nano-drug delivery systems. Few examples of NPs that are applied for piperine delivery include zein-chitosan NPs [30], PLGA NPs [31], and solid-lipid NPs (SLN) [32], etc. Bioperine delivery via poly-lactic acid (PLA)-chitosan-PEG NPs demonstrates the inhibition of P-glycoprotein (P-gp) expression and elevated reactive oxygen species levels to induce cytotoxic effects in multi-drug-resistant breast cancer cells [33]. 

Given that piperine possesses a drug resistance reversal effect, mucin-1 conjugated PEG-PLGA nanoformulation of piperine display superior anti-cancer effects against paclitaxel-resistant MCF-7 breast cancer cells [34]. Previous studies have powerfully illustrated the improvement in curcumin pharmacokinetics by Bioperine by enhancing serum concentration, longer tissue retention, bioavailability, and absorption efficiency [28,35]. We chose to use a PLGA polymer to fabricate the curcumin-Bioperine PLGA nanoparticles (Cur-Bio PLGA NPs) due to their wide application in the delivery of many drugs, including nutraceutical compounds, and their immense biocompatible and biodegradable properties [36]. Moreover, since the translation of nutraceuticals via nano-drug delivery systems is the premise of our present work, the co-delivery of curcumin with a natural bio-enhancer such as Bioperine is an exciting approach as a complementary therapy for atherosclerosis, which will add to the growing list of innovative anti-atherosclerotic treatments.

## 2. Experimental Section

### 2.1. Materials

Poly (D,L-lactic-co-glycolic acid) ester terminated (PLGA) (MW: 7000–17,000, 50:50), polyvinyl alcohol (PVA), MW 31–50 kDa, 87–89% hydrolyzed, and phorbol 12-myristate 13-acetate (PMA) were procured from Sigma-Aldrich, St. Louis, MI, USA. Curcumin and Bioperine were a kind gift from Sami Sabinsa Group, Sami Labs, Bengaluru, India. Organic solvents such as ethyl acetate (EtOAc), dimethyl sulfoxide (DMSO), and ethanol were purchased from Kanto chemicals, Tokyo, Japan. RPMI-1640 medium was from Sigma-Aldrich, St. Louis, MI, USA, fetal bovine serum (FBS) was from Biowest, Nuaillé, France, penicillin (5000 U/mL)/streptomycin (5000 μg/mL), GlutaMAX, and phosphate buffer saline (PBS) were procured from Gibco (Life Technologies, Carlsbad, CA, USA). We procured Alamar blue cell viability kit from Invitrogen and a cholesterol quantitation assay kit from Sigma Aldrich. 1,1′-Dioctadecyl-3,3,3′,3′-tetramethylindocarbocyanine perchlorate acetylated low-density lipoprotein (Dil-AcLDL) and oxidized low-density lipoprotein (Ox-LDL) were purchased from Thermo Fisher Scientific (Waltham, MA, USA). NucBlue Live Ready Probes Reagent was procured from Thermo Fisher Scientific. Applied Biosystems TaqMan Gene Expression assays (FAM-labeled) against the genes CCL2, CD-36, NF-κB, STAT-3, SCAR-B1, GAPDH, were purchased from Thermo Fisher Scientific.

### 2.2. Synthesis of Curcumin-Bioperine Loaded PLGA Nanoparticles (Cur-Bio-PLGA NPs)

We adopted the single emulsion-solvent evaporation method to synthesize the Cur-Bio-PLGA NPs [25]. We synthesized two different formulations of the Cur-Bio-PLGA NPs with the curcumin: Bioperine: PLGA ratios as 1:1:10 and 1:0.2:10, respectively. For example, to synthesize the Cur-Bio-PLGA NPs in the ratio (1:1:10), we took 5 mg of curcumin, 5 mg of Bioperine, and 50 mg of PLGA polymer. While preparing NPs in the ratio (1:0.2:10), 5 mg of curcumin, 1 mg of Bioperine, and 50 mg of PLGA were used. The polymer and the drugs were dissolved in 2 mL of ethyl acetate and kept for 30 min under magnetic stirring (200–500 rpm). The curcumin-Bioperine PLGA organic mixture was then added dropwise to 4 mL of 5% aqueous PVA solution under continuous magnetic stirring (200–500 rpm) followed by sonication at 40 kHz for three minutes. The sonication of the organic–aqueous mixture created a fine emulsion of smaller polymeric droplets. The resulting emulsion was added to 100 mL of 0.3% PVA aqueous solution and stirred rapidly (200–500 rpm) for five hours to aid the evaporation of ethyl acetate. The NPs were collected by centrifuging the above mixture at 8000 rpm for 30 min and washed thrice with Milli Q water. The NPs were freeze-dried and stored at −20 °C until further use. We followed the single emulsion-solvent evaporation procedure to prepare the blank PLGA NPs, curcumin PLGA NPs (Cur-PLGA NPs), and Bioperine PLGA NPs (Bio-PLGA NPs). For blank PLGA NPs, we took 50 mg of PLGA, whereas for the single drug-loaded Cur-PLGA NPs 5 mg of curcumin and 50 mg of PLGA and for the Bio-PLGA NPs 5 mg of Bioperine and 50 mg of PLGA were taken. 

### 2.3. Yield and Encapsulation Studies

Yield efficiency, encapsulation efficiency, and drug loading efficiency were determined using the following formulae.

(1) $$\%\;\mathrm{Yield}\;=\frac{Weight\;of\;dual\;drug\;NPs}{\left(Weight\;of\;Curcumin+Weight\;of\;BioPerine+Weight\;of\;PLGA\right)}\times 100$$

(2) $$\%\;\mathrm{Encapsulation}\;\mathrm{efficiency}=\frac{\mathrm{Ci}-\mathrm{Cf}}{\mathrm{Ci}}\times 100$$

C_i_ is the initial drug concentration, and C_f_ is the concentration of free drug in the supernatant. The amount of free drug in the supernatant was quantified with a standard calibration curve for curcumin and Bioperine [37].

(3) $$\%\;\mathrm{Drug}\;\mathrm{loading}\;\mathrm{efficiency}=\frac{\;Weight\;of\;encapsulated\;drug\;}{Weight\;of\;dual\;drug\;NPs}\times 100\;.$$

### 2.4. Characterization of Nanoparticles

The size and structural morphology of curcumin-BioPerine PLGA NPs were characterized using scanning electron microscopy (SEM) (Hitachi SU-6600) and transmission electron microscopy (TEM) (JEOL’s JEM-2100 FS). For SEM analysis, 5–10 μL of curcumin-BioPerine PLGA NPs dispersed in Milli Q water was dropped onto clean silicon (Si) substrate wafer and vacuum dried. The dried sample was then subjected to a platinum (Pt) sputter coat for 40 s using Hitachi E-1030, Ion sputter. We observed the coated Cur-Bio PLGA NPs under SEM operating at an accelerating voltage of 5 kV.

For TEM imaging, a drop of the NPs sample was deposited on a previously hydrophilized copper (Cu) grid, air-dried at room temperature, and viewed under TEM.

The size and structural morphology of Cur-Bio-PLGA NPs was characterized using atomic force microscopy (AFM). For AFM analysis, 10 μL of NP solution was dropped onto the Si substrate wafer and vacuum dried. AFM utilizes a super sharp tip/probe with a radius of less than 5.0 nm to perform the surface imaging of the NPs (Nanosensors, Asylum Research).

Particle size distribution and the zeta-potential were measured using Zetasizer (Malvern, NanoZs). A disposable sizing cuvette was used for particle size distribution analyses, and the mean diameter and polydispersity index (PDI) values were analyzed and recorded using zeta sizer software. Zeta potential measurements were carried out using a dip cell.

Fourier-transform infrared spectroscopy (ATR-FTIR) (Thermo Scientific, Nicolet iS50 FTIR) was used to investigate the chemical bonding patterns between the two drugs (curcumin and Bioperine) and the PLGA polymer. The spectra were recorded in the range of 4000 to 500 cm^−1^ with a resolution of 4 cm^−1^.

The surface chemistry and elemental composition of the free drugs, blank PLGA NPs, and Cur-Bio-PLGA NPs were analyzed by X-ray photoelectron spectroscopy (XPS, AXIS His-165 Ultra, Kratos Analytical, Shimadzu Corporation, Kyoto, Japan). The binding energy spectra for all the samples were subjected to a survey scan from 0 to 1000 eV with a pass energy of 80 eV and dual magnesium (Mg) as the transmission mode. Curve fitting analyses were carried out using the software provided by the manufacturer. 

The crystalline structures of the free drugs (curcumin and Bioperine) and the Cur-Bio-PLGA NPs were identified by X-ray diffractometry (XRD, Smart Lab, Rigaku-RINT), with a Cu radiation source (Kα = 1.5418 Å) at 45 kV and 200 mA. The scan angle was changed between 5° and 50° with a scan size of 0.02° and a scan rate of 1° min^−1^.

### 2.5. In Vitro Drug Release Studies

For the drug release study, 10 mg of the Cur-Bio-PLGA NPs was dispersed in 10 mL of phosphate buffer saline (PBS) pH 7.4 and divided into 10 Eppendorf tubes, each with a concentration of 500 μg/mL. All tubes were kept at 37 °C in a shaking incubator (150 rpm) for five days. At pre-determined time intervals, the tubes were taken out and centrifuged at a speed of 15,000 rpm for 30 min. The drug concentrations in the supernatant were measured using a UV-Vis spectrophotometer. The absorbance was recorded at λ_430_ for curcumin and λ_342_ for Bioperine to calculate their concentrations in the released samples using a standard curve. The following formula was applied to calculate the percentage of drug release for curcumin and Bioperine [38].

(4) $$\%\;\mathrm{Drug}\;\mathrm{Release}=\frac{Released\;drug\;from\;the\;NPs}{Total\;drug\;encapsulated\;inside\;NPs}\times 100$$

### 2.6. Cell Culture

The human monocytic cell line (THP-1) was purchased from American Type Culture Collection (ATCC), Manassas, VA, USA. The cells were cultured in RPMI-1640 medium supplemented with 10% fetal bovine serum, penicillin (5000 U/mL)/streptomycin (5000 μg/mL), 2 mM GlutaMAX. The cells were grown in 37 °C containing 5% CO_2_ until they reached 80–90% confluency. The cells were sub-cultured every 2 to 3 days.

### 2.7. THP-1 Cell Differentiation and Foam Cell Formation

Three days before conducting cell experiments, THP-1 cell suspension was subjected to centrifugation at 1000 rpm for 7 min, and the cell pellet was resuspended in RPMI-1640 medium containing 200 nM PMA. The cell suspension was then seeded into desired multi-well plates and left for incubation at 37 °C for three days [39]. The cells adhered to the bottom of the culture plates and differentiated into a macrophage-like morphology. We replaced the PMA-containing medium with serum-free fresh RPMI-1640 medium on day three, followed by 24 h resting period. Following the differentiation period, the adherent macrophages were treated with 4 μg/mL Ox-LDL or Dil-AcLDL for 4 h, allowing the formation of foam cells in order to carry out the further cell-based experiments.

### 2.8. In Vitro Cell Viability Studies

The biocompatibility of Cur-PLGA NPs, Bio-PLGA NPs, and Cur-Bio-PLGA NPs on the THP-1 cells was evaluated by Alamar blue fluorometric assay. The assay was performed as per the manufacturer’s protocol. For the assay, THP-1 suspension cells were seeded in a 96-well microtiter plate at a density of 5–6 × 10^3^ cells and differentiated into macrophages as described in Section 2.7. After the macrophage differentiation, the NP suspension (Cur-PLGA NPs, Bio-PLGA NPs, and Cur-Bio PLGA NPs) in RPMI media were added to the cells at different concentrations of 25 μg/mL, 50 μg/mL, 100 μg/mL, 150 μg/mL, 200 μg/mL, and 250 μg/mL. The cells were left for incubation under the same culture conditions for 24, 48 and 72 h. At the end of each incubation period, 10% of Alamar blue dye was added to the cells, incubated for 4 h, and then we recorded the fluorescence at the excitation wavelength (540–570 nm) and emission wavelength (580–610 nm) using a microplate reader (Power scan HT Microplate reader, Dainippon Sumitomo Pharma, Osaka, Japan). The assay was conducted in three independent experiments (triplicates), and the percentage of cell viability was calculated using the formula as given in Equation (5).

(5) $$\%\;\mathrm{Relative}\;\mathrm{Cell}\;\mathrm{Viability}=\frac{A_{test}}{A_{Control}\;}\times 100$$

*A_test_* is the absorbance of the test sample and *A_control_* is the absorbance of the control sample. Untreated cells were taken as positive controls with 100% cell viability. 

### 2.9. Cholesterol Quantitation Assay

A cholesterol quantitation assay determines the concentration of cholesterol present in the foam cells. THP-1 cells were seeded in a 12-well plate at a density of (1 × 10^6^ cells) and differentiated into macrophages as described earlier. The cells were then pre-treated with Cur-PLGA NPs and both the ratios of Cur-Bio PLGA NPs (1:1:10 and 1:0.2:10) at different concentration of 25 μg/mL, 50 μg/mL, 100 μg/mL, 150 μg/mL, 200 μg/mL, and 250 μg/mL. The NP-treated cells were incubated for 24, 48, and 72 h study periods. At the end of each incubation period, the cells were treated with 4 μg/mL Ox-LDL for 4 h and finally washed with PBS. Following the washing steps, the assay was performed according to the manufacturer’s protocol. Relative cholesterol content was calculated using the following formula shown in Equation (6).

(6) $$\mathrm{Relative}\;\mathrm{Cholesterol}\;\mathrm{Content}=\frac{Sa}{Sv}$$

*S_a_* is the amount of cholesterol in a sample obtained from the cholesterol standard curve and *S_v_* is the sample volume.

### 2.10. Inhibition on Foam Cell Formation by Confocal Microscopy

THP-1 cells were seeded in confocal dishes and differentiated into macrophages as described earlier in Section 2.7 to check the inhibitory effect of the Cur-Bio PLGA NPs in the formation of foam cells. We followed two approaches to check the inhibitory activity on the foam cell formation by the Cur-Bio PLGA NPs. In the first approach, the differentiated THP-1 monocyte cells were pre-incubated with 4 μg/mL concentration of Dil-AcLDL for 4 h, followed by treatment with the 1:1:10 and1:0.2:10 ratios of Cur-Bio PLGA NPs (250 μg/mL) for 24 h. The cells were then washed with PBS and stained with NucBlue nuclear dye. The cells were then observed in confocal laser scanning microscopy to analyze the inhibition of foam cell formation. In the second approach, the cells were first pre-treated with the Cur-Bio PLGA NPs (1:1:10 and 1:0.2:10) in the concentration of 250 μg/mL. The treated cells were then incubated for 24, 48, and 72 h study periods under the same culture conditions. At the end of each incubation period, the cells were incubated with 4 μg/mL Dil-AcLDL for 4 h, followed by washing with PBS, and then finally stained with NucBlue nuclear dye. Dil-AcLDL dye has an excitation/emission wavelength of 554 nm/571 nm that emits red fluorescence when observed under confocal laser scanning microscopy.

### 2.11. Cellular Uptake of Dil-AcLDL by Flow Cytometry

THP-1 cells were seeded in a 12-well plate at a density of (1 × 10^6^ cells) and were differentiated into macrophages by PMA treatment as described earlier (Section 2.7). The cells were pre-treated first with Cur-PLGA NPs, and both the ratios of Cur-Bio PLGA NPs (1:1:10 and 1:0.2:10) at a concentration of 250 μg/mL for 24–48 study period under the same culture conditions. At the end of each incubation period, 4 μg/mL of Dil-AcLDL was added to the cells, which were incubated for 4 h, followed by washing with PBS (thrice). The cells were harvested, followed by centrifugation at 1000 rpm for 7 min. Cell fixation was done by treating the cells with 500 μL of 4% paraformaldehyde for 20 min and then washing with PBS. The cells were resuspended in FACS staining buffer and subjected to flow cytometry analysis immediately (BD FACS Canto II, BD Bio Biosciences).

### 2.12. Quantitative Real-Time PCR (qRT-PCR)

Differentiated THP-1 cells at a density of (5 × 10^5^ cells) were pre-treated with both the ratios of Cur-Bio PLGA NPs (1:1:10 and 1:0.2:10) at two concentrations of 100 μg/mL and 250 μg/mL. The NP-treated cells were then incubated for 72 h. Next, the cells were incubated with Ox-LDL for 4 h to induce foam cell formation. The cells were harvested to isolate the total RNA by RNeasy Micro kit and converted into cDNA using Superscript-III reverse transcriptase. The cDNA template was used for qPCR analysis. The qRT-PCR was performed using TaqMan universal master mix, pre-designed TaqMan gene expression primer/probes assay against the genes CD-36, NF-κB, CCL2, STAT-3, SCAR-B1, GAPDH. Real-time quantification system Applied Biosystems 7900 Fast Real-Time PCR system was used for the experiment. The TaqMan gene expression assay IDs of the respective target genes are CD-36: Hs00354519_m1, NF-κB: Hs00765730_m1, CCL2: Hs00234140_m1, STAT-3: Hs00374280_m1, SCAR-B1: Hs00969821_m1, GAPDH: Hs02786624_g1. The relative mRNA expression levels were calculated and normalized relative to GAPDH mRNA as the internal control using the ΔΔCt method.

### 2.13. Statistical Analysis

The data were expressed as the mean ± standard error (S.E.M.). Unpaired Student’s *t*-test was performed using GraphPad Prism. The data were considered significant when *p* < 0.05. 

## 3. Results and Discussion

### 3.1. Characterization of Cur-Bio PLGA NPs

**Figure 1:**
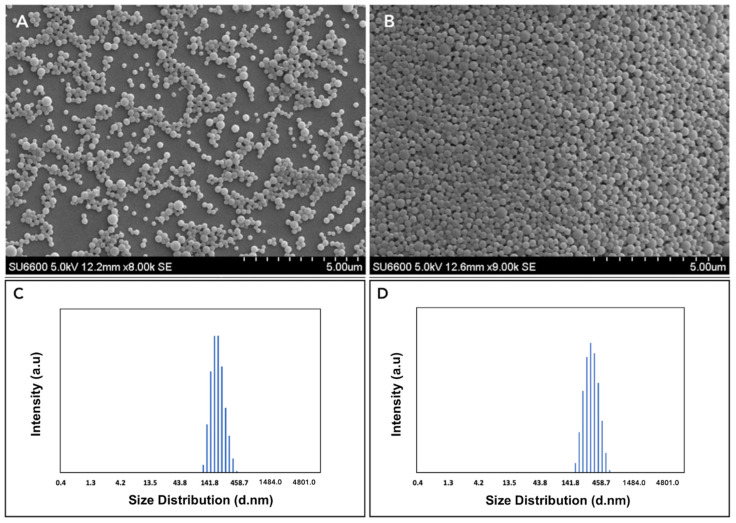
(**A**,**B**) SEM image of curcumin-Bioperine PLGA NPs 1:0.2:10 and 1:1:10 (at scale 5.00 μm). (**C**,**D**) Size distribution of curcumin-Bioperine PLGA NPs 1:0.2:10 and 1:1:10, respectively.

**Figure 2:**
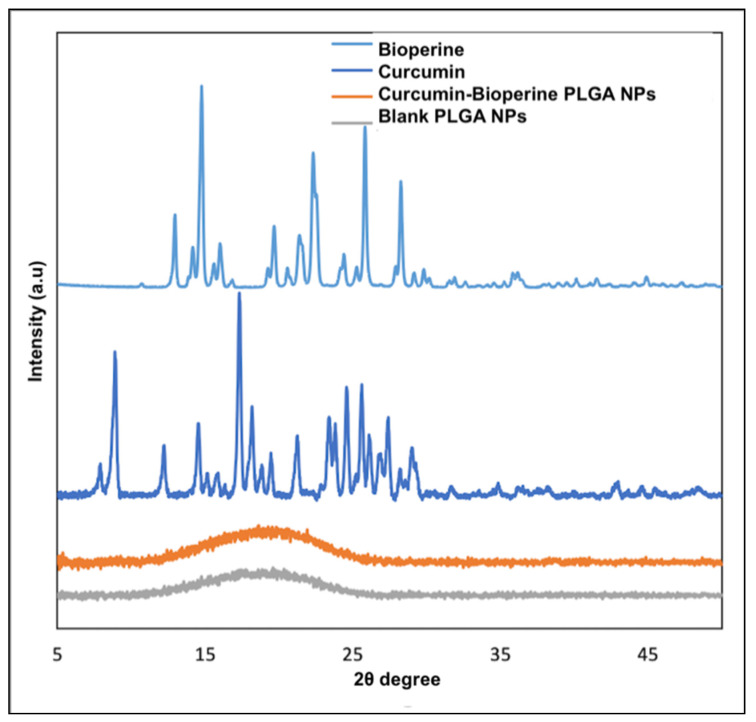
XRD spectra of free Bioperine, free curcumin, curcumin-Bioperine PLGA NPs, and blank PLGA NPs.

As discussed earlier, the fabrication of a PLGA nanoformulation co-encapsulating curcumin and Bioperine improves their bioavailability and absorption efficiency [23,25]. We carried out initial studies of synthesizing Cur-Bio PLGA NPs encapsulating different ratios of curcumin and Bioperine. Based on our optimization studies, we found that from the different ratios of the Cur-Bio PLGA NPs that we developed, NPs of two ratios of curcumin: Bioperine: PLGA (1:1:10 and 1:0.2:10 respectively) displayed monodispersed nanoparticles and high encapsulation efficiencies of the two drugs. Hence, we decided to carry out further characterization studies including in vitro cell studies using only the two ratios of Cur-Bio PLGA NPs (1:1:10 and 1:0.2:10), to support our objective of Bioperine improving curcumin’s efficacy in inhibiting foam cell formation in THP-1 monocyte-derived macrophage cells. The synthesized Cur-Bio PLGA NPs achieved much better aqueous solubility than raw curcumin and Bioperine. The percentage yield of both the Cur-Bio PLGA NPs (1:1:10 and 1:0.2:10), encapsulation (Supplementary Figure S1), and drug loading efficiency of curcumin and Bioperine in both the NPs is shown in Table 1. The high encapsulation efficiency of both drugs could probably be due to PVA usage as a surfactant in the synthesis process [40]. The shape and surface morphology of both the ratios of Cur-Bio PLGA NPs (1:0.2:10 and 1:1:10) revealed a spherical shape and smooth surface, as displayed in SEM images (Figure 1A,B). The size distribution analysis by DLS further confirmed the Cur-Bio PLGA NPs size in the range of 140–450 nm shown in Figure 1C,D. The spherical Cur-Bio PLGA NPs display monodispersity, as evident from SEM results and the PDI values, respectively (Table 1). The uniformity in the size, shape, and smooth surface of the Cur-Bio PLGA NPs was corroborated by TEM and AFM analysis shown in Supplementary Figure S2. The zeta potential for both the Cur-Bio PLGA NPs are shown in Table 1. We analyzed the chemical bonding patterns in the Cur-Bio PLGA NPs by FTIR analysis, which revealed the characteristic peaks of the PLGA polymer at 1745 cm^−1^ (C=O carbonyl stretching) and 1150–1300 cm^−1^ (ester C-O stretching) [41]. The FTIR spectra of Cur-Bio PLGA NPs, as shown in Supplementary Figure S3, did not reveal any significant loss or shift of the functional peaks, concluding that there was no primary drug–polymer interaction. We observed peaks at 3508–3524 cm^−1^ (phenolic O-H stretching) which can be assigned to curcumin [42] (Supplementary Figure S4) and Bioperine at 1580 cm^−1^ (aromatic stretching of benzene ring) (Supplementary Figure S4) [43], 1638 cm^−1^ (–CO–N stretching, C=C diene symmetric and asymmetric stretching) [43]. The surface chemistry analysis of nanoparticles gives out information regarding the elemental composition of the NPs along with the presence of any unwanted contamination during preparation of the NPs. The XPS wide-scan analysis of the blank PLGA NPs and raw curcumin showed carbon 1s (284.6 eV) and oxygen 1s (532 eV) in the spectra shown in Supplementary Figure S5. Bioperine also showed carbon and oxygen peaks and the presence of nitrogen 1 s peak at 398 eV due to a nitrogen atom in its structure. In the Cur-Bio PLGA NPs (1:1:10 and 1:0.2:10), we did not observe any nitrogen peaks on the wide-scan survey implying the successful encapsulation of the drug inside the polymeric matrix. The presence of the carbon and oxygen peaks in the wide-scan spectra of the dual drug NPs might be indicative of the PLGA polymer. XRD analysis provides information regarding the nature of the drugs encapsulated inside the PLGA NPs in a crystalline state or amorphous state. The raw drugs curcumin and Bioperine, as evident from Figure 2, demonstrated a crystalline state, whereas when synthesized into Cur-Bio PLGA NPs, the characteristic peaks of the drugs were absent. This phenomenon of losing the crystallinity of the drugs is a plausible explanation of the intermolecular interaction occurring within the PLGA matrix that leads to the formation of an amorphous state. Similar results were reported regarding the amorphous nature of drugs when encapsulated inside PLGA nanoparticles from XRD analysis showing the uniform drug distribution within the polymeric matrix [44].

### 3.2. In Vitro Curcumin and Bioperine Release from the NPs

**Figure 3:**
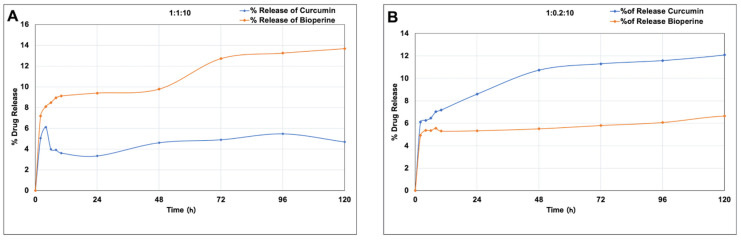
In vitro drug release of curcumin and Bioperine from the curcumin-Bioperine PLGA NPs (**A**) 1:1:10 and (**B**) 1:0.2:10 at pH 7.4 PBS buffer 37 °C for 5 days.

Previous findings elucidate the difference in drug release patterns of dual drug-loaded NPs that can be utilized for therapeutic targeting of two different targets simultaneously [45]. In this work, we observed that both the ratios of Cur-Bio PLGA NPs demonstrated a slight difference in curcumin and Bioperine release patterns. Upon investigation, we found that in a 1:1:10 ratio, Cur-Bio PLGA NPs showed a faster and gradual increased release of Bioperine from 7.2 to 15.33% throughout the study period of 5 days. At the same time, curcumin had a comparatively slower release profile of only 3–5%, as shown in Figure 3A. We speculated the reason behind this phenomenon could be due to the equal concentration of both the drugs entrapped inside the PLGA NPs, where Bioperine release could be affected due to its small molecular size. Feng S. et al. carried out such studies to demonstrate the drug release profile of three different model drugs (BSA, lysozyme, and vancomycin) where release rates were affected by their molecular weight, amongst other multiple factors [46]. Curcumin release rates are also influenced by the surface coating of nanoparticles, as reported by Hasan M. et al. [47]. Uncoated curcumin-loaded nanoliposomes with a curcumin concentration of 0.2 mg/mL tend to have a faster-sustained release profile of 22–24%. The biphasic release profile of curcumin from the chitosan-coated nanoliposomes was positively affected, and was less than 15% at the same curcumin concentration of 0.2 mg/mL. Nonetheless, both the coated and uncoated curcumin-loaded nanoliposomes with 0.1 mg/mL curcumin concentration had a low release rate of only 10%, suggesting that a lower curcumin concentration encapsulated inside the NPs might have contributed to the low release rate. Moreover, in both the coated and uncoated nanoliposomes, the curcumin release percentage reached only 25% after the study period. This study also lays importance on the fact that the release profile of any encapsulated material inside nanoparticles could be affected significantly by a surface coating of polymers [47].

The 1:0.2:10 ratio Cur-Bio PLGA NPs release profile showed a somewhat relatively faster and sustained release profile of curcumin (8.59–12.08%) in comparison to Bioperine throughout the study period, as shown in Figure 3B. We concluded that the low concentration of Bioperine inside the Cur-Bio PLGA NPs matrix might have contributed to the quicker release pattern of curcumin compared to the 1:1:10 nanoformulation. Curcumin release is also affected when it is co-encapsulated with another drug inside PLGA NPs shown in a study conducted by Prabhuraj R.S. and colleagues [48]. The curcumin release from the curcumin-niclosamide-loaded PLGA NPs reached a sustained release percentage of 20–25% throughout the study period compared to niclosamide [48]. In our previous work, we discussed the low-sustained release pattern of curcumin when co-encapsulated with GANT61 inside PLGA NPs [23]. The difference in dual drug release profiles from polymeric NPs is affected by multiple factors, such as the unique architecture of nanocarriers, physical properties of the drug, drug concentration, drug–polymer interactions, drug–drug interactions, pH of the buffers, and stability in buffers [49]. The different amounts of drug encapsulated inside NPs affecting drug release profiles also have been reported and studied extensively [45]. The advantage of incorporating drugs inside NPs is to have a controlled release profile that will ensure to maintain a required therapeutic concentration for carrying out specified biological actions without exerting any side effects. Henceforth, it observed that even though the release percentage of curcumin is less than in the present study, it still managed to inhibit the foam cell formation in the THP-1 cells, discussed in the in vitro cell studies sections.

### 3.3. In Vitro Cell Viability Studies

**Figure 4:**
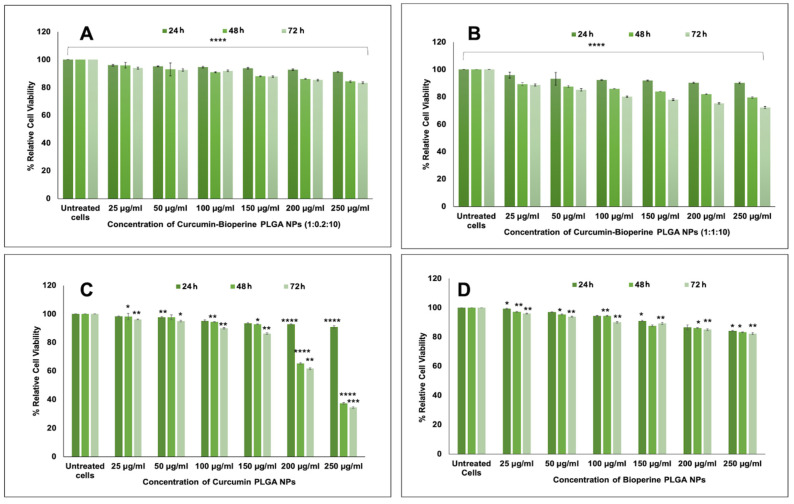
(**A**) **%** Relative cell viability of THP-1 monocyte-derived macrophage cells when treated with 1:0.2:10 ratio curcumin-Bioperine PLGA NPs for 24–72 h. (**B**) **%** Relative cell viability of THP-1 monocyte-derived macrophage cells when treated with 1:1:10 ratio curcumin-Bioperine PLGA NPs for 24–72 h. (**C**) % Relative cell viability of THP-1 monocyte-derived macrophage cells when treated with curcumin PLGA NPs for 24–72 h. (**D**) % Relative cell viability of THP-1 monocyte-derived macrophage cells when treated with Bioperine PLGA NPs for 24–72 h. (All the data for cell viability studies are expressed as the mean ± S.E.M. Unpaired Student’s *t*-test was performed to check the statistical significance of the investigation using GraphPad Prism software. Data were considered significant when *p* < 0.05. Untreated samples were taken as controls). The ratios of curcumin-Bioperine PLGA NPs at all doses and time points demonstrated that the experiment was extremely statistically significant, with *p* < 0.05 (* *p* ≤ 0.05 ** *p* ≤ 0.01 *** *p* ≤ 0.001 **** *p* ≤ 0.0001).

We carried out cell viability studies to study the cytotoxicity of the single-drug NPs (Cur-PLGA NPs and Bio-PLGA NPs) and Cur-Bio PLGA NPs against the THP-1 monocytic cell line. As observed in the cell viability studies (Figure 4A,B), both the ratios (1:0.2:10 and 1:1:10) of Cur-Bio PLGA NPs did not elicit any significant cytotoxicity towards the THP-1 monocyte-derived macrophage cells, suggesting the biocompatible nature of the NPs. The data are extremely statistically significant, with *p* < 0.05 in both the ratios of Cur-Bio PLGA NPs. We also observed that the THP-1 monocyte-derived macrophage cells, when treated with Cur-PLGA NPs, displayed some percentage of inhibitory activity at concentrations 200 μg/mL and 250 μg/mL after 48–72 h, as shown in Figure 4C. The cytotoxic nature of Cur-PLGA NPs suggests that the percentage release of curcumin from the PLGA NPs could be relatively more than when compared with the Cur-Bio PLGA NPs, and the release rate of curcumin might not be affected by the presence of an additional drug inside the polymeric matrix. However, the Bio-PLGA NPs did not elicit any cytotoxicity towards the THP-1 monocyte-derived macrophage cells at all concentrations and times, as shown in Figure 4D. Administration of curcumin has previously demonstrated convincing results against the protection of atherosclerotic lesions via the reduction in lipid, cholesterol, and inflammatory responses [50]. The incorporation of Bioperine as a bio-enhancer in the formulation of Cur-Bio PLGA NPs could be a plausible paradigm shift in the arsenal of existing treatment modalities for atherosclerotic lesions. We concluded that our Cur-Bio PLGA NPs at their highest concentration of 250 μg/mL were non-toxic towards the THP-1 monocyte-derived macrophage cells from our cell viability studies. The non-toxic behavior of both the ratios of Cur-Bio PLGA NPs towards the THP-1 cells is due to the lesser percentage release of curcumin, which might not have reached the inhibitory concentration causing any cytotoxicity towards the THP-1 monocyte-derived macrophage cells but enough to inhibit the target genes and proteins. Min K. et al. studied the inhibitory effect of curcumin on the modulation of CD-36 expression on Ox-LDL-treated RAW 264.7 murine macrophages at a concentration of 10 μM without eliciting any cytotoxicity towards the macrophage cells [51]. Several studies have also reported that different concentrations of curcumin produce different effects on the target gene expression depending on the cell type [52,53,54].

### 3.4. Cholesterol Quantitation Assay

**Figure 5:**
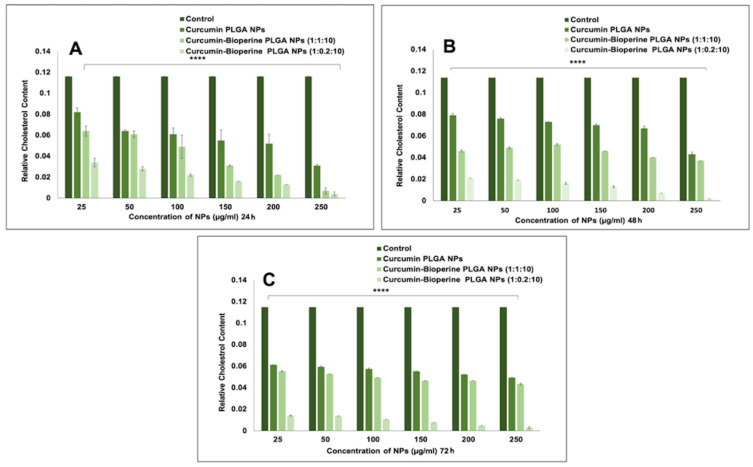
Relative cholesterol content of THP-1 monocyte-derived macrophage cells when treated with curcumin PLGA NPs, and both the ratios of curcumin-Bioperine PLGA NPs (1:1:10 and 1:0.2:10) for (**A**) 24 h (**B**) 48 h and (**C**) 72 h. (All the data for cholesterol quantitation studies are expressed as the mean ± S.E.M. Unpaired Student’s *t*-test was performed to check the statistical significance of the investigation using GraphPad Prism software. Data were considered significant when *p* < 0.05. Untreated samples were taken as controls). The cholesterol quantitation experiment demonstrated extremely statistically significant results, with *p* < 0.05 (**** *p* = 0.0001).

Hyperlipidemia is a crucial risk factor in the induction and promotion of atherosclerosis, where low-density lipoprotein deposits cholesterol in tissue, creating plaque and clogging the coronary arteries [55]. An essential therapeutic intervention for preventing atherosclerosis is to control cholesterol levels. Cholesterol efflux is the rate-limiting step of reverse cholesterol transport. Henceforth, cholesterol quantitation analysis will provide an idea of whether the pre-treatment of THP-1 monocyte-derived macrophage cells with Cur-Bio PLGA NPs will exert a protective effect on the THP-1 monocyte-derived macrophage cells from forming foam cells and controlling cholesterol levels. In this assay, cholesterol esterase detects total cholesterol by a coupled enzyme assay to generate a colored product directly proportional to the amount of cholesterol present in the sample. As shown in Figure 5A–C, the relative cholesterol content in the THP-1 monocyte-derived macrophage cells when pre-treated with 1:1:10 and 1:0.2:10 Cur-Bio PLGA NPs demonstrated a gradual reduction at all concentrations and time points. The 1:0.2:10 ratio of the Cur-Bio PLGA NPs displayed an improved reduction in the cholesterol content in contrast to the 1:1:10 ratio of the Cur-Bio PLGA NPs. We believe this phenomenon might have resulted from the better curcumin release from the 1:0.2:10 Cur-Bio PLGA NPs, suggesting a lesser concentration of Bioperine can also enhance the therapeutic efficacy of curcumin. The Cur-Bio PLGA NPs displayed statistical significance for both the ratios at all concentrations and time points (*p* < 0.05). Cur-PLGA NPs did not significantly reduce the relative cholesterol levels at all concentrations and time in this study compared to Cur-Bio PLGA NPs (Figure 5A–C), which establishes that curcumin efficacy in treating hyperlipidemia could be substantially improved when administered with a bio-enhancer like Bioperine. The cholesterol quantitation study delineates the possibility that the relative cholesterol content decreased in the THP-1 monocyte-derived macrophage cells is due to the rapid cholesterol efflux mediated via the scavenger receptor, class B type 1 (SR-B1) upregulation [54]. SR-B1 is one of the passive mechanisms employed by macrophages to facilitate cholesterol efflux and its upregulation by curcumin via different mechanisms like heme-oxygenase (HO)-1 and ATP-binding cassette transporter (ABCA1) pathways [56] will result in significant outcome to cholesterol efflux that in turn helps to prevent foam cell and plaque formation, thereby halting the atherosclerotic disease progression.

### 3.5. Inhibition on Foam Cell Formation by Confocal Microscopy

**Figure 6:**
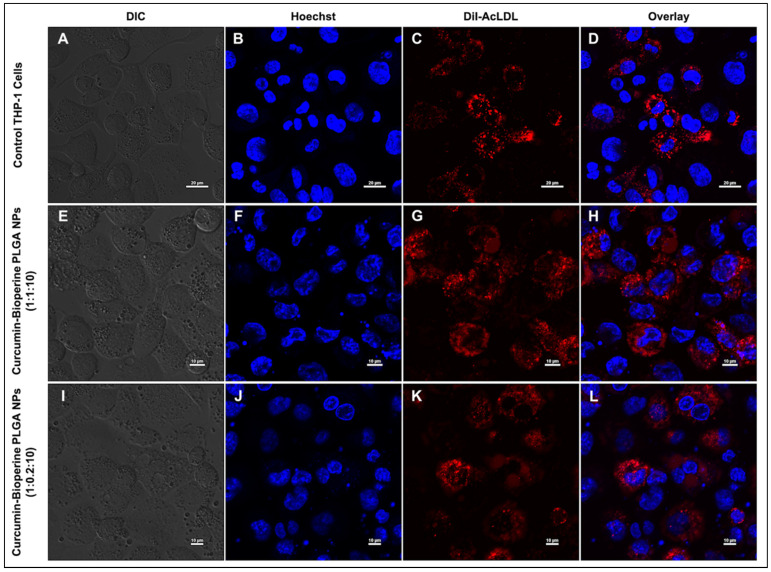
Confocal laser scanning microscopy analysis shows the Dil-AcLDL staining on THP-1 monocyte-derived macrophage cells to check the inhibition of foam cell formation. THP-1 monocyte-derived macrophage cells are pre-incubated with Dil-AcLDL for 4 h, followed by the treatment with both the ratios of curcumin-Bioperine PLGA NPs (1:1:10 and 1:0.2:10) for 24 h. The top panel (**A**–**D**) shows the control THP-1 monocyte-derived macrophage cells forming foam cells (scale bar 20 μm). The second panel (**E**–**H**) shows the THP-1 monocyte-derived macrophage foam cells treated with curcumin-Bioperine PLGA NPs 1:1:10 (Scale bar 10 μm). The third panel (**I**–**L**) shows the THP-1 monocyte-derived macrophage foam cells treated with curcumin-Bioperine PLGA NPs 1:0.2:10 (scale bar 10 μm).

**Figure 7:**
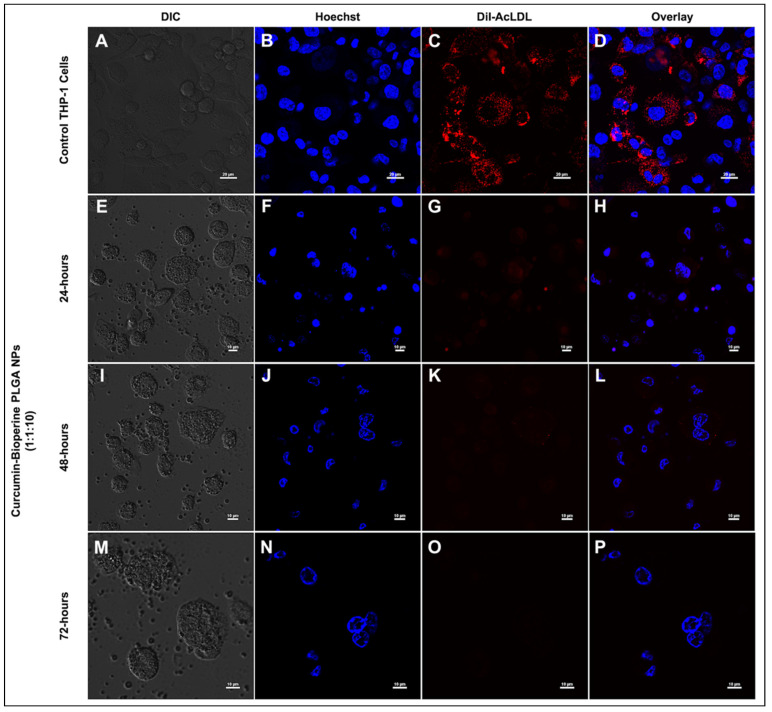
Confocal laser scanning microscopy analysis shows the Dil-AcLDL staining on THP-1 monocyte-derived macrophage cells to check the inhibition of foam cell formation. THP-1 monocyte-derived macrophage cells were pre-treated with curcumin-Bioperine PLGA NPs (1:1:10) for 24–72 h, followed by incubation of Dil-AcLDL for 4 h. The top panel (**A**–**D**) shows the control THP-1 monocyte-derived macrophage cells forming foam cells (Scale bar 20 μm). The second panel (**E**–**H**), third panel (**I**–**L**), and fourth panel (**M**–**P**) show the inhibition of foam cell formation upon treated with curcumin-Bioperine PLGA NPs 1:1:10 for 24–72 h (scale bar 10 μm).

**Figure 8:**
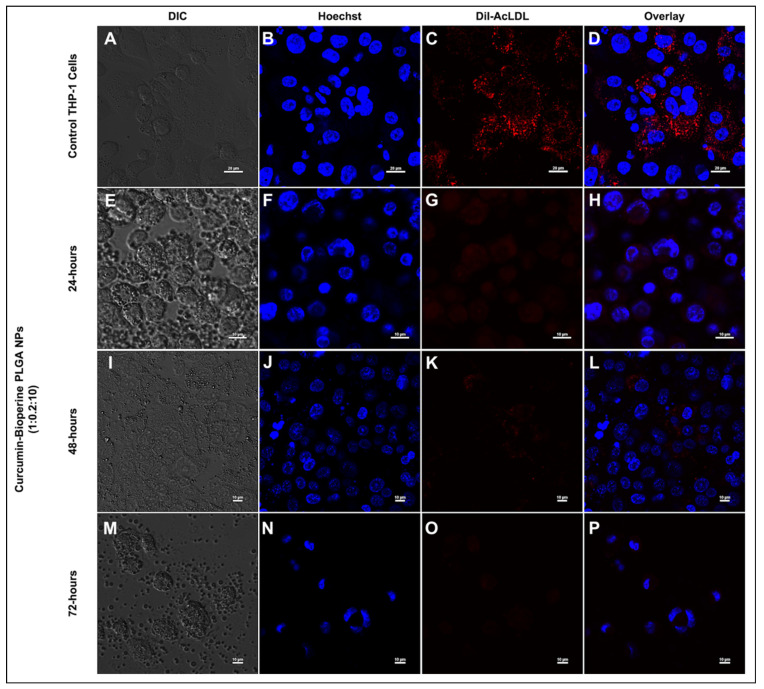
Confocal laser scanning microscopy analysis shows the Dil-AcLDL staining on THP-1 monocyte-derived macrophage cells to check the inhibition of foam cell formation. THP-1 monocyte-derived macrophage cells are pre-treated with curcumin-Bioperine PLGA NPs (1:0.2:10) for 24–72 h, followed by incubation of Dil-AcLDL for 4 h. The top panel (**A**–**D**) shows the control THP-1 monocyte-derived macrophage cells forming foam cells (Scale bar 20 μm). The second panel (**E**–**H**), third panel (**I**–**L**), and fourth panel (**M**–**P**) show the inhibition of foam cell formation upon being treated with curcumin-Bioperine PLGA NPs 1:0.2:10 for 24–72 h (scale bar 10 μm).

The formation of macrophage foam cells is a hallmark event in early-stage atherosclerosis. The uptake of Ox-LDL/Ac-LDL by the macrophages via the expression of scavenger receptors (CD-36 and SR-A1) accumulates lipid in intracellular spaces, forming foam cells that finally trigger signaling cascades leading to the inflammatory response [8]. Previous findings elucidated the inhibitory activity on foam cell formation by curcumin [51,54]. We sought to check the same by our Cur-Bio PLGA NPs on the foam cell forming ability using THP-1 monocyte-derived macrophage cells. We managed to inhibit the foam cell formation by the Cur-Bio PLGA NPs using two strategies. In the first strategy, the THP-1 monocyte-derived macrophage cells were subjected to Dil-AcLDL treatment to form foam cells and then administered with both the ratios (1:1:10 and 1:0.2:10) of Cur-Bio PLGA NPs at a concentration of 250 μg/mL for 24 h to check the inhibition on foam cell formation. As shown in Figure 6A–D, control cells displayed foam cell formation, and from Figure 6E–L, we observed that there was no reduction in the foam cell formation by both the ratios of Cur-Bio PLGA NPs. In the second strategy, we initially treated the THP-1 monocyte-derived macrophage cells with both the ratios (1:1:10 and 1:0.2:10) of Cur-Bio PLGA NPs (250 μg/mL) for 24 h, followed by the administration of Dil-AcLDL for 4 h to induce the formation of foam cells, as shown in Figure 7A,B and Figure 8A,B**.** In this approach, we observed that there was a marked reduction in the formation of foam cells within 24 h of pre-treatment with both the ratios of Cur-Bio PLGA NPs as shown in Figure 7E–H and Figure 8E–H, which could be due to the downregulation of the scavenger receptors involved in the uptake of Dil-AcLDL. Since the second strategy showed promising results in the inhibition of foam cell formation within 24 h of Cur-Bio PLGA NPs (1:1:10 and 1:0.2:10) pre-treatment, we speculated a further decrease within 48–72 h of the NPs treatment. Hence, we experimented with an additional 48–72 h time points to analyze further reduction in the foam cell formation and we observed that the foam cell formation notably reduced, as evident from the decreased Dil-AcLDL fluorescence intensity in the THP-1 monocyte-derived macrophage cells shown in Figure 7I–P and Figure 8I–P. We thus postulate that the administration of Cur-Bio PLGA NPs is an excellent preventive measure that can provide better protection against the development of atherosclerotic plaques.

### 3.6. Cellular Uptake of Dil-AcLDL by Flow Cytometry

**Figure 9:**
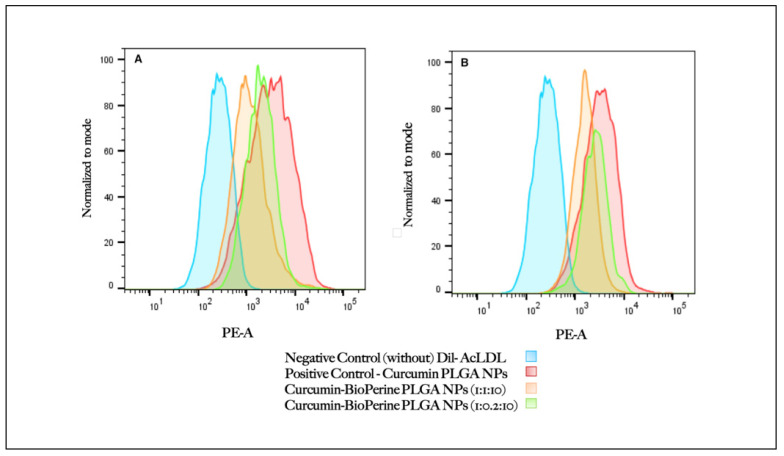
Cellular uptake of Dil-AcLDL by THP-1 monocyte-derived macrophage cells treated with curcumin-Bioperine PLGA NPs (1:1:10 and 1:0.2:10) and Cur-PLGA NPs for (**A**) 24 h, (**B**) 48 h followed by incubation with Dil-AcLDL for 4 h and flow cytometry analysis.

As we have already established from our confocal microscopy results that both the ratios of Cur-Bio PLGA NPs (1:1:10 and 1:0.2:10) at 250 µg/mL are effective in inhibiting the Dil-AcLDL uptake by the THP-1 monocyte-derived macrophage cells within 24–48 h, we decided to study the internalization of the Dil-AcLDL by the macrophage cells using flow cytometry analysis. THP-1 monocyte-derived macrophages were pre-treated with the Cur-Bio PLGA NPs at both ratios at 250 μg/mL for 24–48 h, followed by incubation with Dil-AcLDL for 4 h to analyze the reduction in the LDL uptake. Inhibition of forming foam cells by curcumin is due to the downregulation of CD-36-mediated Ox-LDL uptake [51] and upregulation of ABCA1-dependent cholesterol efflux [57], as reported previously. Flow cytometry analysis revealed that during 24–48 h of treatment with the Cur-Bio PLGA NPs, both 1:1:10 and 1:0.2:10 ratios displayed a similar reduction in the Dil-AcLDL uptake as shown in Figure 9A,B. As evident from our confocal microscopy results that suggest reduced Dil-AcLDL uptake by the THP-1 monocyte-derived macrophage cells from 24–48 h, the flow cytometry analysis also revealed comparable results, showing that both the ratios of Cur-Bio PLGA NPs could block the internalization of Dil-AcLDL by the macrophages within 24–48 h. Notably, even though both the ratios of Cur-Bio PLGA NPs have different concentrations of Bioperine encapsulated inside PLGA NPs, their bio-enhancing capacity was not altered, which helped curcumin to carry out the designated functions of reducing plaque formation in atherosclerosis. Cur-PLGA NPs were used as a positive control and were observed to have an increased uptake of Dil-AcLDL by the THP-1 monocyte-derived macrophage cells compared to the Cur-Bio PLGA NPs, thus suggesting that Bioperine increases curcumin efficacy in minimizing the Dil-AcLDL uptake in THP-1 monocyte-derived macrophage cells.

### 3.7. Gene Expression

**Figure 10:**
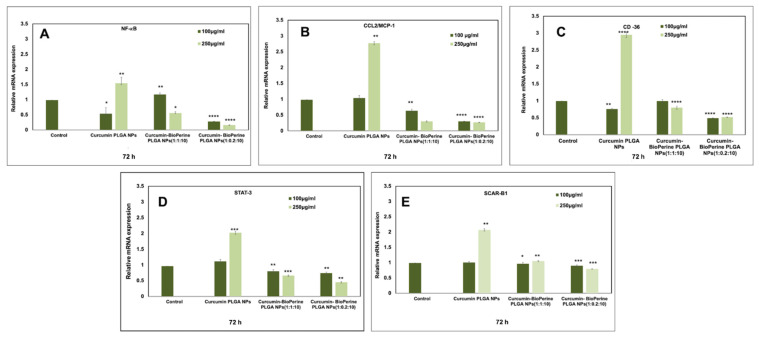
Relative mRNA expression of NF-κB, CCL2/MCP-1, CD-36, STAT-3, and SCAR-B1 in THP-1 monocyte-derived macrophage cells after treatment with curcumin-Bioperine PLGA NPs (1:1:10 and 1:0.2:10) and Cur-PLGA NPs for 72 h. Both the ratios of curcumin-Bioperine PLGA NPs (250 μg/mL) demonstrated downregulation of (**A**) NF-κB, (**B**) CCL2/MCP-1, (**C**) CD-36, (**D**) STAT-3 and upregulation of (**E**) SCAR-B1 compared to 100 μg/mL. All the data were normalized to GAPDH mRNA expression using the ΔΔCt method. (All the data for gene expression studies are expressed as the mean ± S.E.M. Unpaired Student’s *t*-test was performed to check the statistical significance of the investigation using GraphPad Prism software. Data were considered significant when *p* < 0.05 (* *p* ≤ 0.05 ** *p* ≤ 0.01 *** *p* ≤ 0.001 **** *p* ≤ 0.0001). Untreated samples were taken as controls.

Atherosclerotic plaque formation causes various molecular changes in the foam cells, leading to unfavorable conditions inciting cholesterol influx, activation of inflammatory cytokines, and their associated pathways, such as NF-κB [58], which promotes endothelial cell activation by increasing the expression of VCAM-1, ICAM-1, P- and E-selectin, and pro-inflammatory cytokines. These inflammatory cytokines, in turn, activate the STAT-3 pathway that leads to macrophage polarization and inflammation-induced disease pathogenesis [17,59]. In this study, we decided to analyze the relative mRNA expression levels of the genes NF-κB, CCL2/MCP-1, CD-36, STAT-3 and SR-B1/SCAR-B1, implicated to play functional roles in the disease’s progression. Downregulating these genes would substantially reduce the foam cell formation in the arterial wall, leading to reduced plaque build-up. From our confocal microscopy results, we have found out that both the ratios of Cur-Bio PLGA NPs at 250 µg/mL at 72 h showed promising results in inhibiting the foam cell formation in the THP-1 monocyte-derived macrophage cells. Hence, we sought to check whether the Cur-Bio PLGA NPs would elicit downregulating effects on the target genes at that concentration and time. We also decided to include a mid-concentration (100 µg/mL) of the Cur-Bio PLGA NPs for both the ratios (1:1:10 and 1:0.2:10) only for this experiment to do a comparative analysis. THP-1 monocyte-derived macrophage was pre-treated with 100 µg/mL and 250 µg/mL of 1:1:10 and 1:0.2:10 ratio of Cur-Bio PLGA NPs for 72 h, followed by Ox-LDL treatment for 4 h to induce foam cell formation. As shown in Figure 10A, both the ratios of Cur-Bio PLGA NPs at 250 µg/mL were successful in downregulating the NF-κB gene expression compared to the 100 µg/mL concentration of Cur-Bio PLGA NPs and Cur-PLGA NPs. NF-κB’s blockade would help regulate atherosclerosis by reducing foam cell formation, vascular inflammation, and plaque disruption. In a study conducted by Plotkin J.D. et al., fullerene derivatives prevented atherosclerotic lesions in apolipoprotein E null mice via inhibition of foam cell formation by modulating the NF-κB pathway [60]. CCL-2/MCP-1, one of the critical chemokines, promotes the migration and macrophage infiltration to initiate foam cell formation, and its downregulation mediated by the Cur-Bio PLGA NPs will prevent such steps (Figure 10B). The relative gene expression data highlight the successful downregulation of CD-36 by both the ratios of Cur-Bio PLGA NPs at 250 µg/mL comparatively better than 100 µg/mL NPs concentration, as shown in Figure 10C. Dose-dependent administration of curcumin in Ldr^−/−^ rats has been shown to alleviate the adverse side effects of fatty diet on dyslipidemia, inflammatory cytokines expression, and atherosclerosis. This study further reported that the suppression of CD-36 by curcumin is one of the mechanisms employed to modulate atherogenesis [61]. The established findings of curcumin inhibiting the genes (CCL-2/MCP-1 and CD-36) implicated in atherosclerosis have been discussed extensively in the past [18,51]. Henceforth, our studies in preventing foam cell formation in atherosclerosis via modulating NF-κB pathway, CD-36, and CCL-2/MCP-1 genes corroborate previous experimental findings. The Cur-Bio PLGA NPs successfully regulate STAT-3 expression, as shown in Figure 10D, which is involved in the production of cytokines, dysregulated expression of adhesion molecules, and growth factors promoting atherosclerosis progression [17]. Since atherosclerotic plaque often faces hypercholesterolemia due to increased cholesterol esters accumulation in the macrophages, its removal by SR-B1/SCAR-B1 will facilitate the reduction in foam cells. There exist four mechanisms of cholesterol efflux, namely, aqueous diffusion, facilitated diffusion by SR-B1, active unidirectional efflux by ABCA1, and ABCG1 [62]. Curcumin is known to remove excess cholesterol by upregulating SR-B1/SCAR-B1 and is positively supported by the Cur-Bio PLGA NPs, as shown in Figure 10E. This upregulation of SR-B1/SCAR-B1 by the Cur-Bio PLGA NPs in a dose- and time-dependent manner indicates successful cholesterol efflux from the THP-1 monocyte-derived macrophage cells, which also supports the cholesterol quantitation study. In this experiment, we found that the ratio of 1:0.2:10 Cur-Bio PLGA NPs (250 µg/mL) at 72 h demonstrated consistent results in the gene expression analysis, with greater statistical significance (*p* < 0.05) than the 1:1:10 ratio of Cur-Bio PLGA NPs and Cur-PLGA NPs. Cur-PLGA NPs, as observed in Figure 10A–E, displayed inconsistent results in regulating the target genes. Henceforth, we conclude that the incorporation of Bioperine could substantially improve the therapeutic efficacy of curcumin to induce downregulation and upregulation of the targeted genes involved in atherosclerotic foam cell formation.

## 4. Conclusions

In this study looking at preventing plaque growth, a promising phytonutrients-based nanomedicine, Cur-Bio PLGA NPs, demonstrated anti-atherosclerotic activity by reducing foam cell formation in THP-1 monocyte-derived macrophage cells in an in vitro setting. We have presented a novel nano-nutraceutical-based Cur-Bio PLGA NP, investigating the bio-enhancing capacity of Bioperine to improve the therapeutic efficacy of curcumin towards the inhibition of foam cell formation in atherosclerosis. Two different ratios of Cur-Bio PLGA NPs (1:1:10 and 1:0.2:10) were selected after optimization studies to explore their size, shape, surface chemistry, chemical interaction, nature of the drugs, and in vitro cell studies. Both the ratios of the Cur-Bio PLGA NPs (1:1:10 and 1:0.2:10) had spherical morphology, with an average size of 295.3 nm and 181 nm, respectively. We concluded that both the drugs were successfully encapsulated inside the PLGA NPs without showing any primary drug–polymer interactions from the different characterization studies. We observed that the 1:0.2:10 ratio of Cur-Bio PLGA NPs had an increased percentage release profile of curcumin compared to the 1:1:10 ratio. We also noticed the biocompatibility of the Cur-Bio PLGA NPs towards the THP-1 monocyte-derived macrophage cells at all concentrations and time points. In terms of cholesterol quantitation assay, the 1:0.2:10 ratio of Cur-Bio PLGA NPs exhibited a decrease in cholesterol content in the THP-1 monocyte-derived macrophage cells which was comparatively better than the 1:1:10 ratio at all concentrations and time points, with 250 μg/mL having the best outcome in reducing cholesterol. From the remaining cell studies, both the ratios of Cur-Bio PLGA NPs demonstrated the inhibition of foam cell formation, cellular uptake of Dil-AcLDL, and expression of the target genes involved in atherosclerosis plaque formation, where 1:0.2:10 Cur-Bio PLGA NPs displayed improved results. This study’s findings posit that curcumin therapeutic efficacy in decelerating atherosclerotic foam cell formation is improved by the co-delivery of bio-enhancers such as Bioperine via nano-drug delivery systems. Nano-nutraceutical formulations could be administered as a complementary therapy with the current line of atherosclerotic treatment options for a heightened therapeutic benefit. Additional extensive studies need to be conducted in the future to figure out the dosage, duration, and pharmacokinetic/pharmacodynamic analysis with the help of metabolomics studies before their clinical translation. Nano-nutraceutical formulations like Cur-Bio PLGA NPs envision a promising role as a protective and preventive strategy to tackle alarming cardiovascular diseases despite their onset at any age.

## Figures and Tables

**Table 1:** Physico–chemical characterizations of curcumin-Bioperine PLGA Nanoparticles.

Curcumin-Bioperine PLGA Nanoparticles	Particle Size (nm)	PDI ^a^	ZP ^b^ (mV)	% EE ^c^		% YE ^d^	% LE ^e^	
				Curcumin	Bioperine		Curcumin	Bioperine
1:1:10	293 ± 4.15	0.01 ± 0.004	−21 ± 0.88	97.4 ± 0.44	98.2 ± 0.03	55 ± 1.73	16.25 ± 0.3	15.67 ± 0.68
1:0.2:10	181 ± 8.63	0.21 ± 0.005	−17.6 ± 0.33	98.2 ± 1.01	85.8 ± 0.41	49.6 ± 0.88	17.9 ± 1.2	17.2 ± 0.008

^a^ Poly Dispersity Index (PDI), ^b^ Zeta Potential (mV), ^c^ % Encapsulation Efficiency, ^d^Yield Efficiency, ^e^ % Loading Efficiency.

## Data Availability

Not applicable.

## Supplementary Materials

The following are available online at https://www.mdpi.com/article/10.3390/pharmaceutics13091420/s1, Figure S1: UV-Vis spectra of Cur-Bio PLGA NPs showing the encapsulation of Bioperine at λ_342_ and Curcumin at λ_430_., Figure S2: 3D AFM images of Curcumin-Bioperine PLGA NPs (A) 1:0.2:10 and (B) 1:1:10. TEM images of Curcumin-Bioperine PLGA NPs (C) 1:0.2:10 and (D) 1:1:10 (At scale 50 nm), Figure S3: FTIR analysis of free Curcumin, free Bioperine, Blank PLGA NPs, Curcumin-PLGA NPs, Bioperine-PLGA NPs, and Curcumin-Bioperine PLGA NPs in the range of 4000 to 500 cm^−1^, Figure S4: FTIR spectra of Curcumin-Bioperine PLGA NPs showing the characteristic peaks of curcumin (3508 cm^−1^) and Bioperine (1580 cm^−1^), Figure S5: XPS Spectra of free Curcumin, free Bioperine, Blank PLGA NPs, and Curcumin-Bioperine PLGA NPs (1:0.2:10 and 1:1:10). The nitrogen (N 1s) peak present in free Bioperine is shown in the inlet box. (Si2p and Si2s peaks have arisen from the SiO_2_ substrate).
