# Supplementary material for: Co-Delivery of Curcumin and Bioperine via PLGA Nanoparticles to Prevent Atherosclerotic Foam Cell Formation
Source: Pharmaceutics. 2021 Sep 8;13(9):1420. doi: 10.3390/pharmaceutics13091420 (PMC8468720; doi:10.3390/pharmaceutics13091420)
Supplement: Supplementary file 1 [file pharmaceutics-13-01420-s001.zip › pharmaceutics-1335449-supplementary.pdf]

pharmaceutics

MDPI

Supplementary Materials: Co-Delivery of Curcumin and Bioperine via PLGA Nanoparticles to Prevent Atherosclerotic Foam Cell Formation

Sindhu C Pillai, Ankita Borah, Minh Nguyen Tuyet Le, Hiroaki Kawano, Kouichi Hasegawa and D. Sakthi Kumar

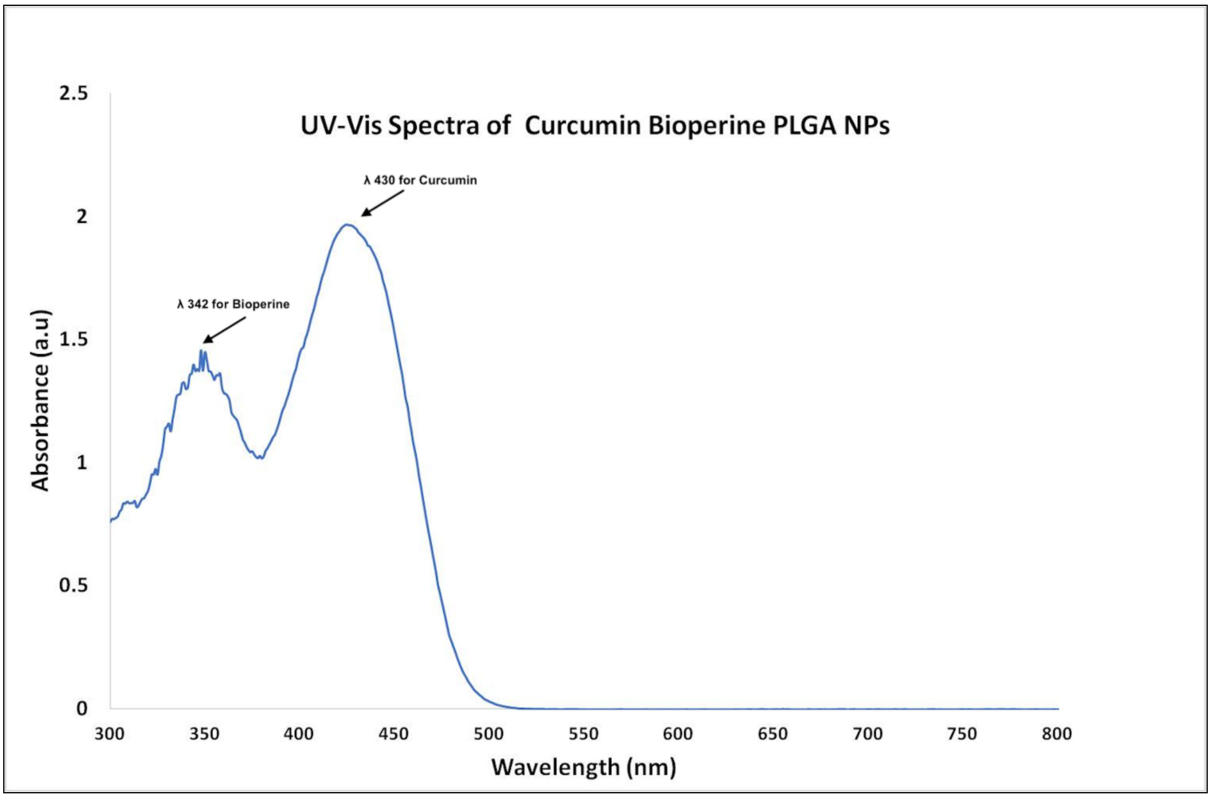

Figure S1. UV-Vis spectra of Cur-Bio PLGA NPs showing the encapsulation of Bioperine at λ_{342} and Curcumin at λ_{430}.

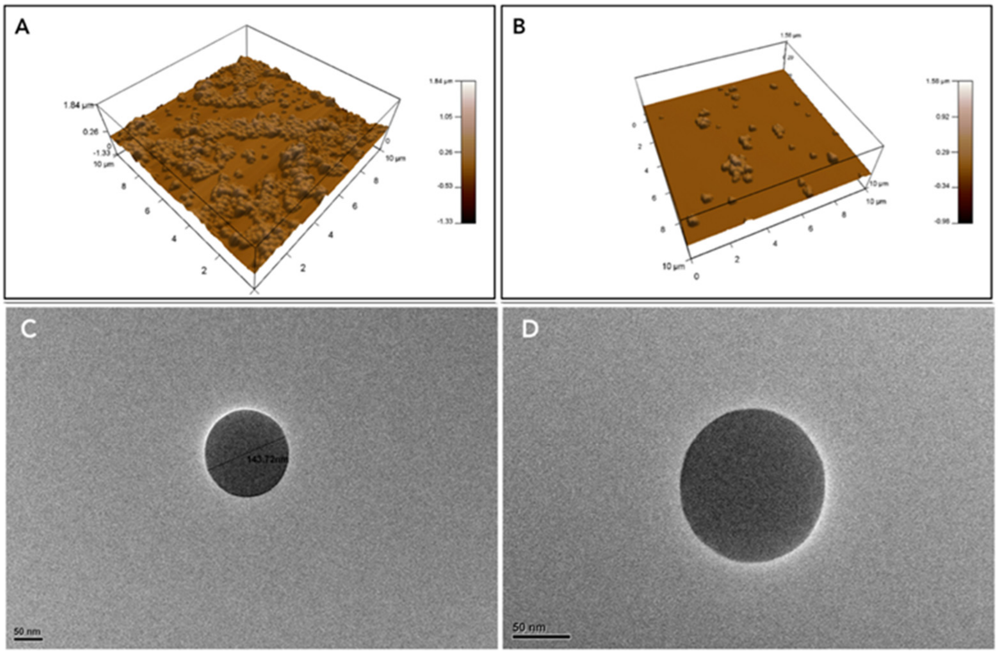

Figure S2. 3D AFM images of Curcumin-Bioperine PLGA NPs (A) 1:0.2:10 and (B) 1:1:10. TEM images of Curcumin-Bioperine PLGA NPs (C) 1:0.2:10 and (D) 1:1:10 (At scale 50 nm).

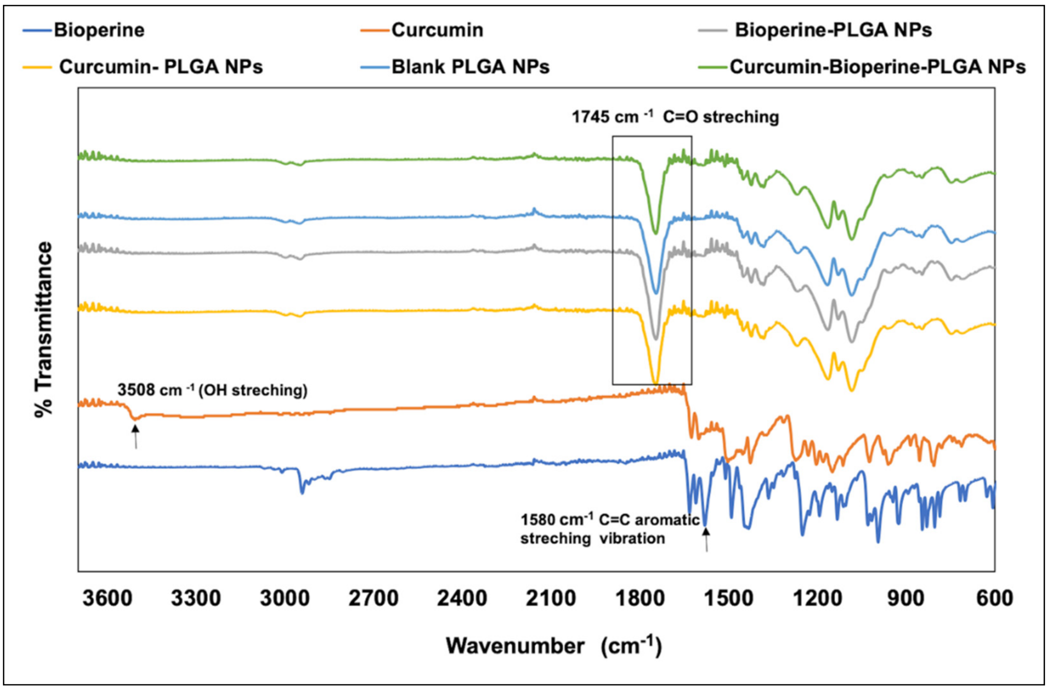

Figure S3. FTIR analysis of free Curcumin, free Bioperine, Blank PLGA NPs, Curcumin-PLGA NPs, Bioperine-PLGA NPs, and Curcumin-Bioperine PLGA NPs in the range of 4000 to 500 cm^{-1}.

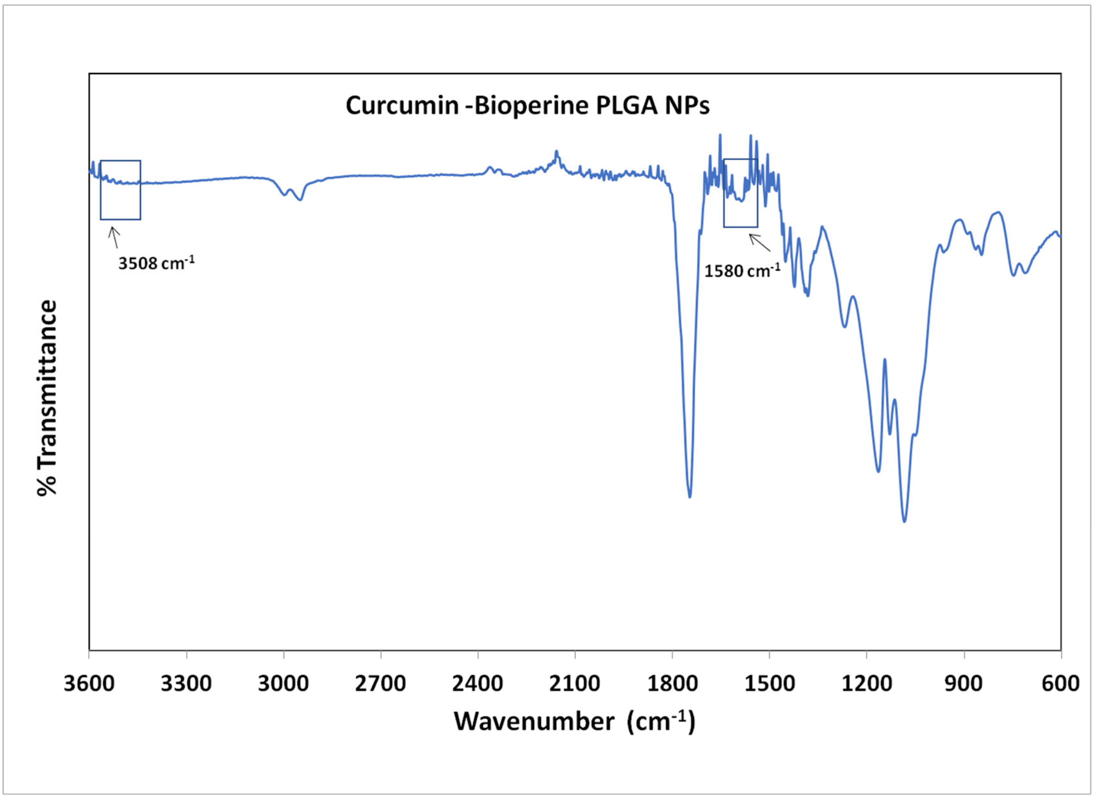

Figure S4. FTIR spectra of Curcumin-Bioperine PLGA NPs showing the characteristic peaks of curcumin (3508 cm^{-1}) and Bioperine (1580 cm^{-1}).

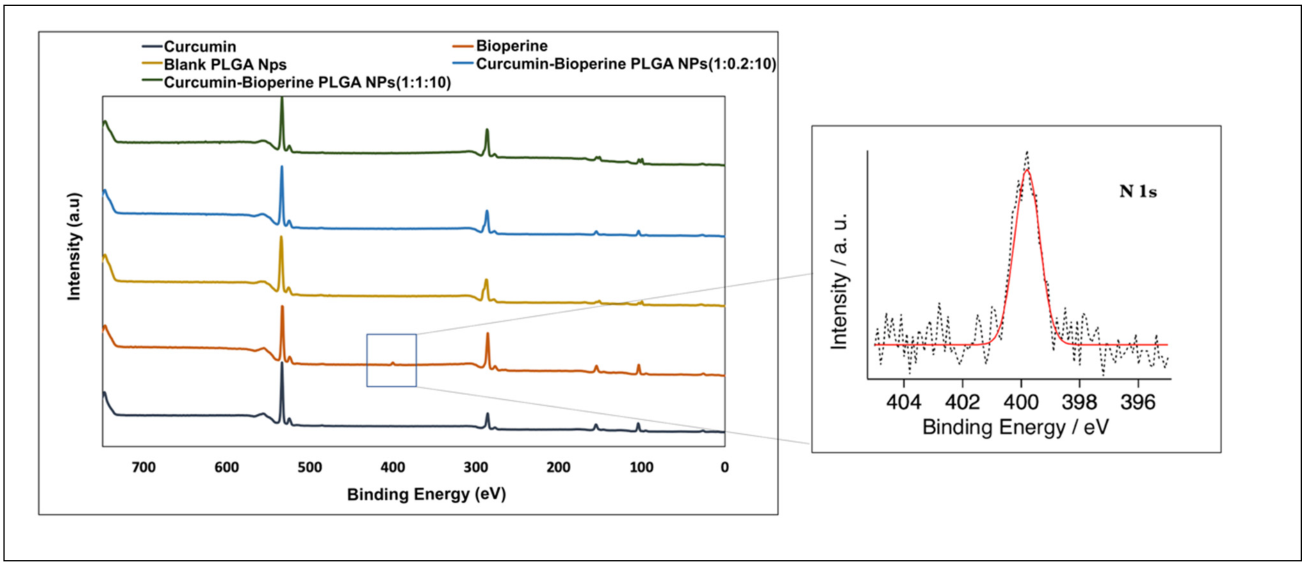

Figure S5. XPS Spectra of free Curcumin, free Bioperine, Blank PLGA NPs, and Curcumin-Bioperine PLGA NPs (1:0.2:10 and 1:1:10). The nitrogen (N 1s) peak present in free Bioperine is shown in the inlet box. (Si2p and Si2s peaks have arisen from the SiO2 substrate).
